# Antiproliferative, Cytotoxic, and Antimigratory Effects of Licochalcone A in Ishikawa Endometrial Cancer Cells

**DOI:** 10.3390/ph19101611

**Published:** 2026-10-10

**Authors:** Tuğba Ekiz-Yılmaz, Fatma Beyza Sağ

**Affiliations:** Department of Histology and Embryology, Istanbul Faculty of Medicine, Istanbul University, Istanbul 34093, Turkey; beyzasag@istanbul.edu.tr

**Keywords:** Licochalcone A, endometrial cancer, Ishikawa cells, cell proliferation, cell death, migration, Akt/mTOR

## Abstract

**Background/Objectives**: Licochalcone A (LA), a bioactive chalcone derived from *Glycyrrhiza* species, exhibits anticancer activity in several tumor models; however, its effects on endometrial cancer remain poorly understood. This study investigated the antiproliferative, cytotoxic, and antimigratory effects of LA in Ishikawa endometrial cancer cells and characterized associated changes in Akt/mTOR- and LC3-related molecular responses. **Methods:** Metabolic activity, colony formation, cell cycle distribution, migration, and invasion were evaluated following LA treatment. Ki-67 and LC3-II immunoreactivity, PI3K, Akt, mTOR, and MAP1LC3B transcript levels, and Akt phosphorylation were also assessed. **Results:** LA reduced metabolic activity and clonogenic capacity, decreased Ki-67 expression after prolonged exposure, increased apoptosis, and induced S-phase accumulation at higher concentrations. LA markedly reduced cell migration after 48 h, whereas no statistically significant change in invasion was detected. RT-qPCR revealed concentration- and time-dependent changes in PI3K, Akt1, mTOR, and MAP1LC3B transcript levels, with mTOR significantly reduced at all tested concentrations after 48 h. Akt phosphorylation was markedly reduced across all tested concentrations after 48 h, while MAP1LC3B transcript levels and LC3-II immunoreactivity showed concentration- and time-dependent changes. **Conclusions:** These findings demonstrate antiproliferative, cytotoxic, and antimigratory effects of LA in Ishikawa endometrial cancer cells and identify associated changes in Akt/mTOR- and LC3-related molecular responses.

## 1. Introduction

Endometrial cancer (EC) is one of the most common malignancies of the female reproductive system and remains a major cause of gynecological cancer-related mortality [1]. Its association with metabolic disorders, including obesity and type 2 diabetes mellitus, further contributes to its growing public health burden [2]. For advanced or recurrent EC, carboplatin plus paclitaxel represents the standard first-line chemotherapy regimen [3]. More recently, immune checkpoint inhibitors, including pembrolizumab and dostarlimab, have been incorporated into chemotherapy-based treatment strategies and have significantly improved progression-free survival in patients with advanced or recurrent EC [3,4]. However, these systemic treatments are associated with clinically relevant adverse events, including nausea, fatigue, alopecia, peripheral neuropathy, and anemia, as well as immune-related adverse events associated with immune checkpoint inhibition [3,4]. These treatment-related toxicities, together with the need for continued therapeutic improvement, underscore the importance of exploring novel compounds with potential anticancer activity [3,4]. Advances in understanding the molecular pathogenesis of EC have increased interest in molecularly targeted therapies, highlighting the need to identify novel compounds that modulate signaling pathways involved in tumor development and progression [2,5].

Natural products have emerged as important sources of bioactive compounds with potential anticancer properties [6]. Licochalcone A (LA), a naturally occurring chalcone derived from licorice root, has attracted considerable attention because of its diverse bio-logical activities, including antitumor, antiangiogenic, antioxidant, and anti-inflammatory effects [7]. Given the importance of altered cellular metabolism and proliferative signaling in tumor growth, compounds that target multiple cancer-associated processes are of particular interest [8]. Accumulating evidence indicates that LA can interfere with multiple aspects of malignant behavior, including cell proliferation, survival, cell cycle progression, migration, and invasion [9,10].

The phosphoinositide 3-kinase (PI3K)/Akt/mammalian target of rapamycin (mTOR) signaling axis is a major regulator of cell growth, proliferation, metabolism, survival, and therapeutic resistance and is frequently dysregulated in human cancers [11,12,13,14,15,16]. Aberrant signaling through this axis is also relevant to endometrial cancer and has been implicated in resistance to therapy-induced apoptosis [13,15]. Autophagy is a fundamental cellular process that maintains intracellular homeostasis by lysosomally degrading damaged organelles and unnecessary biomolecules [17]. In EC, alterations in autophagy have been implicated in tumor cell proliferation and therapeutic response, particularly in the development of progestin resistance [18]. Experimental studies in Ishikawa cells have shown a close link between autophagy and PI3K/Akt/mTOR signaling, where activation of this pathway promotes cell proliferation and progestin resistance by suppressing autophagy [18]. Conversely, inhibition of PI3K/Akt/mTOR signaling has been shown to enhance autophagy-related responses and suppress EC cell growth, further supporting the functional relevance of autophagy in EC [18,19]. These observations make PI3K/Akt/mTOR and autophagy-related molecular responses particularly relevant when evaluating the anticancer activity of bioactive compounds.

Previous studies have linked LA’s anticancer effects to modulation of PI3K/Akt/mTOR signaling and autophagy-related responses. LA has been reported to inhibit this signaling axis and induce apoptosis and autophagy in breast and cervical cancer cells [20,21], while LC3-II-related changes have also been described following LA treatment [22]. Moreover, LA reduces cell viability and clonogenic growth and alters cell cycle progression in several tumor models, including osteosarcoma and gastric cancer [9,23,24]. More recently, LA has also been reported to reduce cell viability and induce apoptosis in endometrial cancer cells through GRP78-mediated endoplasmic reticulum stress [25]. However, the broader cellular effects of LA and their association with Akt/mTOR and LC3-related molecular responses remain insufficiently characterized in Ishikawa endometrial cancer cells. Therefore, the present study aimed to investigate the effects of LA on clonogenic capacity, proliferation, cell cycle progression, apoptosis, migration, and invasion in Ishikawa endometrial cancer cells and to characterize associated changes in PI3K/Akt/mTOR and LC3-related molecular responses.

## 2. Results

### 2.1. Licochalcone A Reduces Metabolic Activity in Ishikawa Cells

**Figure 1:**
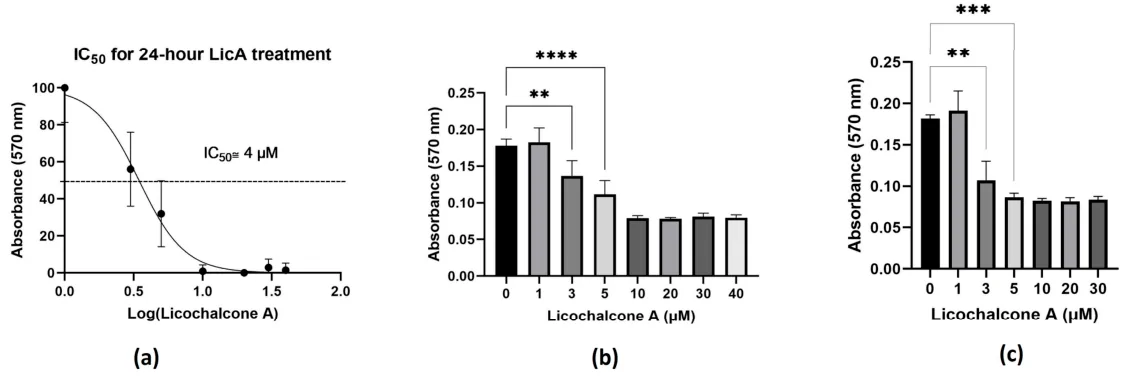
Effect of LA on the metabolic activity of Ishikawa cells. (**a**) Nonlinear dose–response curve used to determine the IC_50_ value (4 µM) following 24 h of LA treatment. (**b**) Absorbance-based assessment of metabolic activity following treatment with increasing concentrations of LA (0–40 µM) for 24 h and (**c**) LA (0–30 µM) for 48 h. Data are presented as mean ± SD from three independent experiments (n = 3). ** *p* < 0.01, *** *p* < 0.001, and **** *p* < 0.0001.

The effect of LA on the metabolic activity of Ishikawa cells was evaluated using the MTT assay. Ishikawa cells were exposed to increasing concentrations of LA (0, 1, 3, 5, 10, 20, 30, and 40 µM) for 24 and 48 h. However, in the 48 h experiment, 40 µM dose group was excluded because no measurable value was obtained. Following 24 h of exposure, LA reduced metabolic activity in a concentration-dependent manner compared with the untreated control group (Figure 1a,b). A significant reduction was observed at 3 µM (*p* < 0.01), with further significant decreases at concentrations of 5 µM and above (*p* < 0.0001). The IC_50_ value following 24 h of LA exposure was approximately 4 µM (95% confidence interval: 2.9–4.1 µM), as determined using variable-slope nonlinear regression (Figure 1a). Based on the 24 h IC_50_ value, LA concentrations of 4, 8, 15, and 30 µM were selected for subsequent experiments.

To further evaluate the effect of prolonged LA exposure, metabolic activity was additionally assessed after 48 h. LA treatment significantly reduced metabolic activity at 3 µM (*p* < 0.01) and at 5, 10, 20, and 30 µM (*p* < 0.001) compared with the untreated control group (Figure 1c). These findings demonstrate a reduction in MTT assessed metabolic activity following both 24 and 48 h of LA exposure.

### 2.2. LA Suppresses Colony Formation in Ishikawa Cells

**Figure 2:**
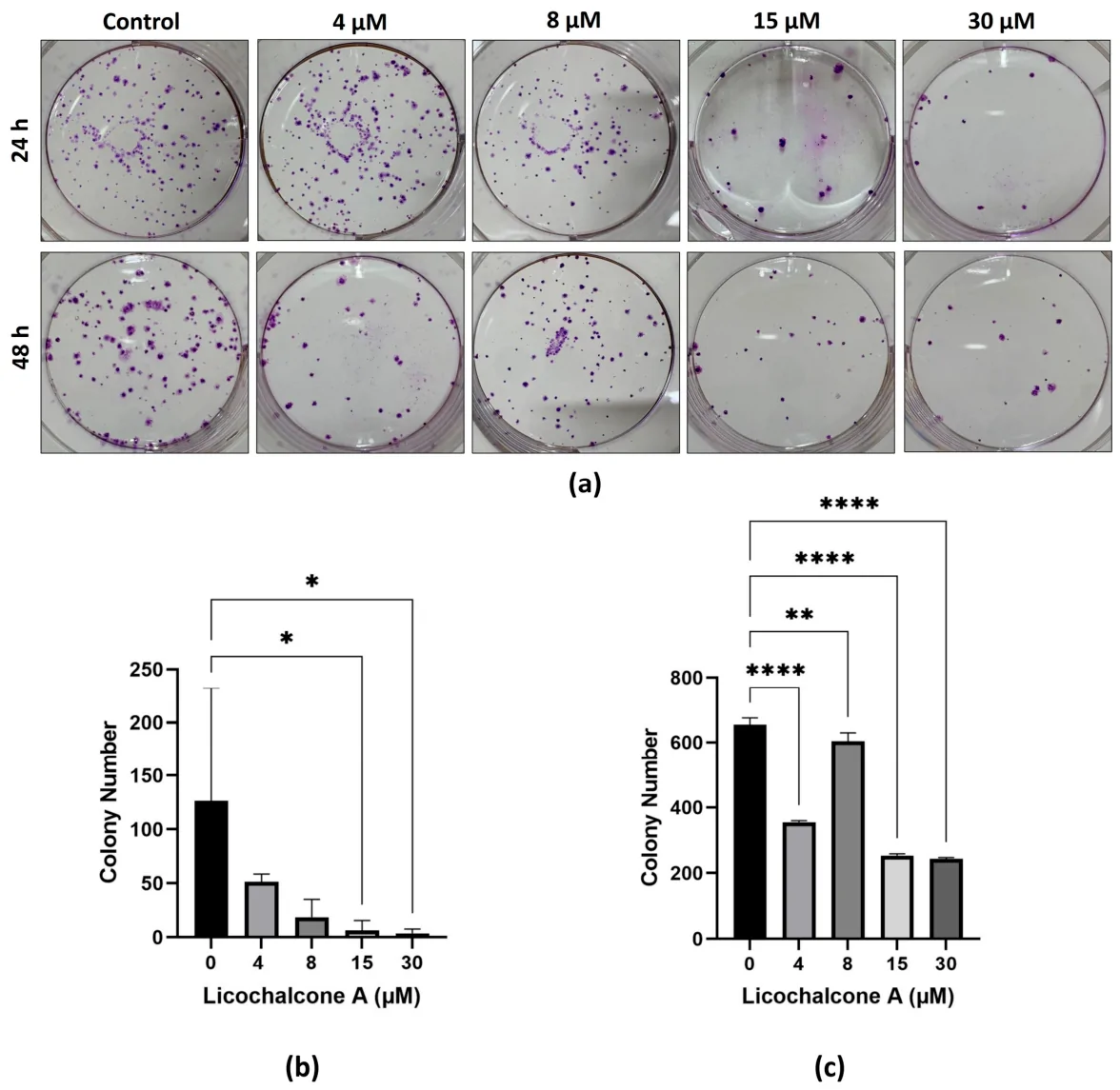
Effect of LA on colony formation in Ishikawa cells. (**a**) Representative macroscopic images of colony formation well plates following treatment with LA (0, 4, 8, 15, and 30 µM) for 24 and 48 h. Quantification of colony numbers following (**b**) 24 h and (**c**) 48 h of LA treatment. Data are presented as mean ± SD from three independent experiments (n = 3). * *p* < 0.05, ** *p* < 0.01, and **** *p* < 0.0001.

Colony formation assays were carried out to investigate further the effect of LA on the colony-forming capacity of Ishikawa cells following treatment with LA (0, 4, 8, 15, and 30 µM) for 24 and 48 h (Figure 2a). After 24 h of exposure, LA reduced colony formation in a concentration-dependent manner compared with the untreated control. Although lower concentrations (4 and 8 µM) showed a trend toward decreased colony formation, inhibition was statistically significant at 15 and 30 µM (*p* < 0.05 for both; Figure 2b). Following 48 h of LA treatment, colony formation was significantly reduced at all concentrations tested compared with the untreated control. Statistical analysis showed significant reductions at 4, 15, and 30 µM (*p* < 0.0001), and 8 µM (*p* < 0.01; Figure 2c). These findings demonstrate that LA suppresses the clonogenic capacity of Ishikawa cells in a concentration and time-dependent manner.

### 2.3. LA Increases Apoptosis in Ishikawa Cells

**Figure 3:**
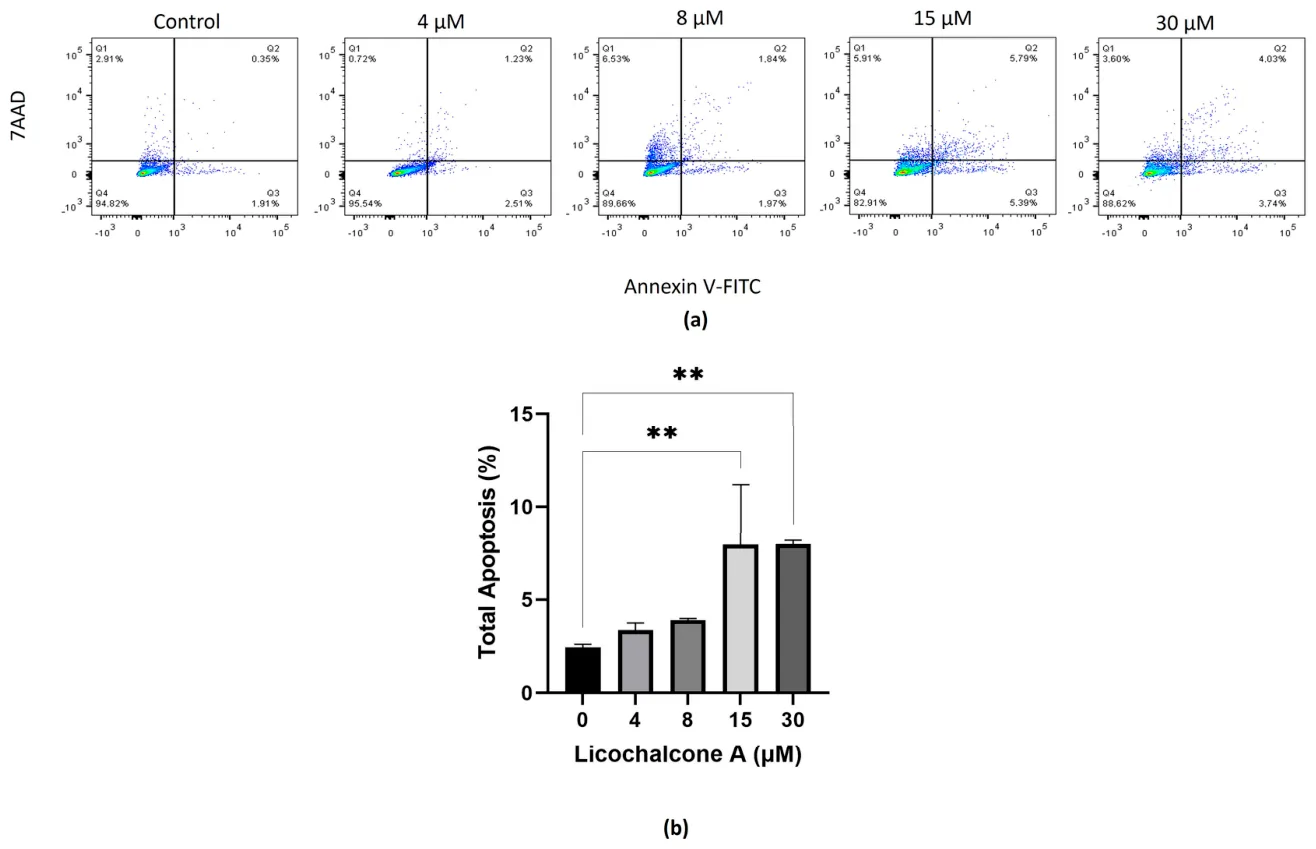
Effect of LA on apoptosis in Ishikawa cells. (**a**) Representative Annexin V-FITC/7-AAD flow cytometry plots of Ishikawa cells following treatment with LA (0, 4, 8, 15, and 30 µM) for 24 h. (**b**) Quantitative analysis of total apoptosis (the sum of early and late apoptotic cell populations). Data are presented as the mean ± SD from three independent experiments (n = 3). ** *p* < 0.01.

To evaluate LA’s effect on apoptosis, Ishikawa cells were treated with increasing concentrations of LA (0, 4, 8, 15, and 30 µM) for 24 h and analyzed by Annexin V-FITC/7-AAD flow cytometry (Figure 3a). Total apoptosis (the sum of early and late apoptotic cell populations) increased significantly at 15 µM (*p* = 0.0066) and 30 µM (*p* = 0.0064) compared with the control group, whereas the increases at 4 and 8 µM were not statistically significant (Figure 3b).

### 2.4. Effect of LA on Cell Cycle Distribution in Ishikawa Cells

**Figure 4:**
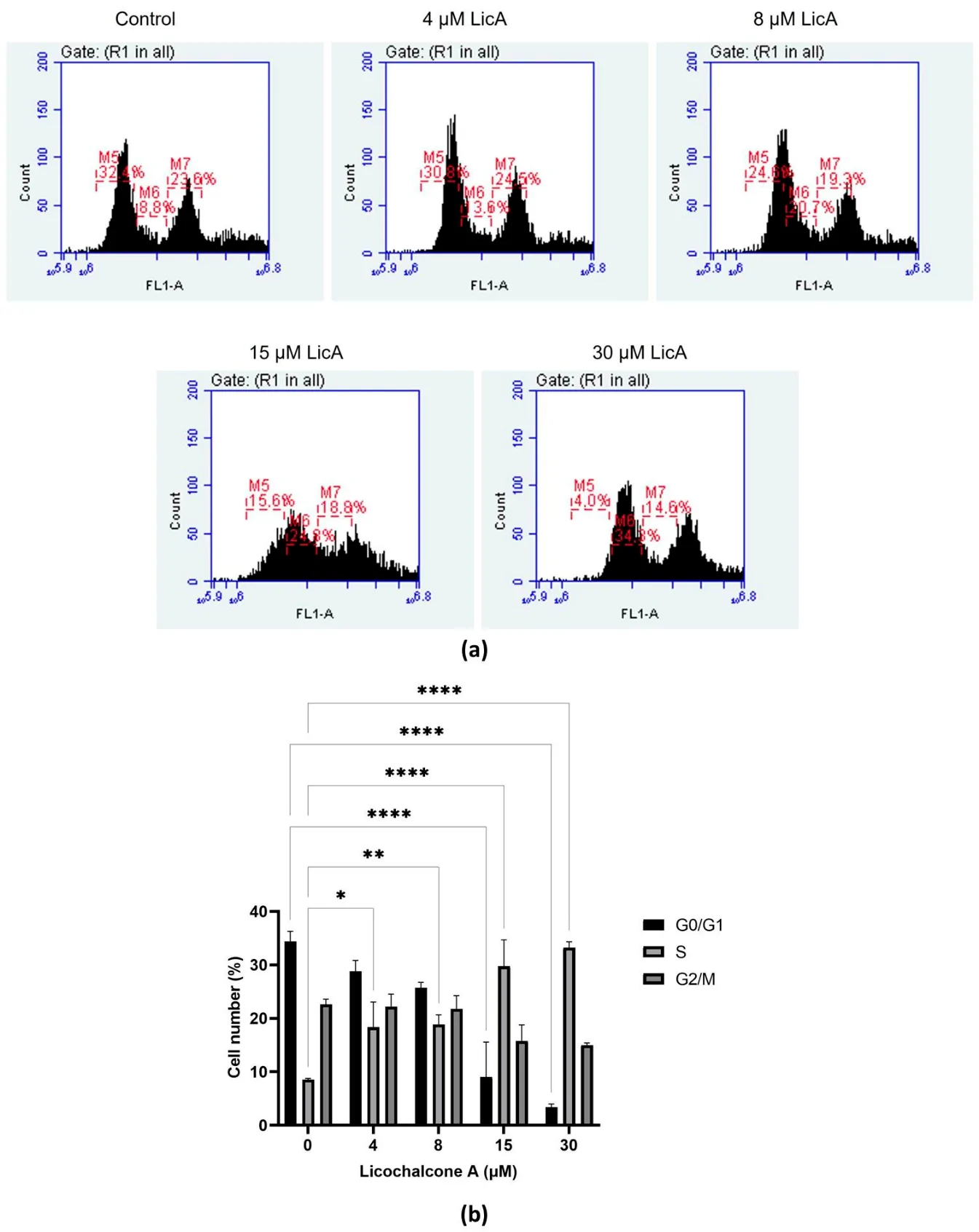
Effect of LA on cell cycle distribution in Ishikawa cells. (**a**) Representative flow cytometric DNA-content histograms of Ishikawa cells treated with LA (0, 4, 8, 15, and 30 µM) for 24 h, showing the regions assigned to G0/G1 (M5), S (M6), and G2/M (M7) phases. (**b**) Quantitative analysis of the percentage of cells in each cell cycle phase (G0/G1, S, and G2/M) following LA treatment. Data are presented as the mean ± SD from three independent experiments (n = 3). * *p* < 0.05, ** *p* < 0.01, and **** *p* < 0.0001.

The effect of LA on cell cycle progression was assessed by flow cytometry following treatment of Ishikawa cells with increasing concentrations of LA (0, 4, 8, 15, and 30 µM) for 24 h (Figure 4a). LA treatment induced concentration-dependent alterations in cell cycle distribution. At lower concentrations, the proportion of cells in the S phase was significantly increased at 4 µM (*p* = 0.0158) and 8 µM (*p* = 0.0093), whereas no significant changes were observed in the G0/G1 or G2/M phases. In contrast, higher concentrations of LA (15 and 30 µM) markedly reduced the proportion of cells in the G0/G1 phase (*p* < 0.0001 for both), accompanied by a pronounced accumulation of cells in the S phase (*p* < 0.0001 for both; Figure 4b). No significant changes were detected in the G2/M phase at any LA concentration. These findings indicate that LA alters cell cycle progression primarily by promoting S-phase accumulation, with a marked reduction in the G0/G1 population at higher concentrations.

### 2.5. LA Suppresses Ki-67 Expression Following Prolonged Exposure

**Figure 5:**
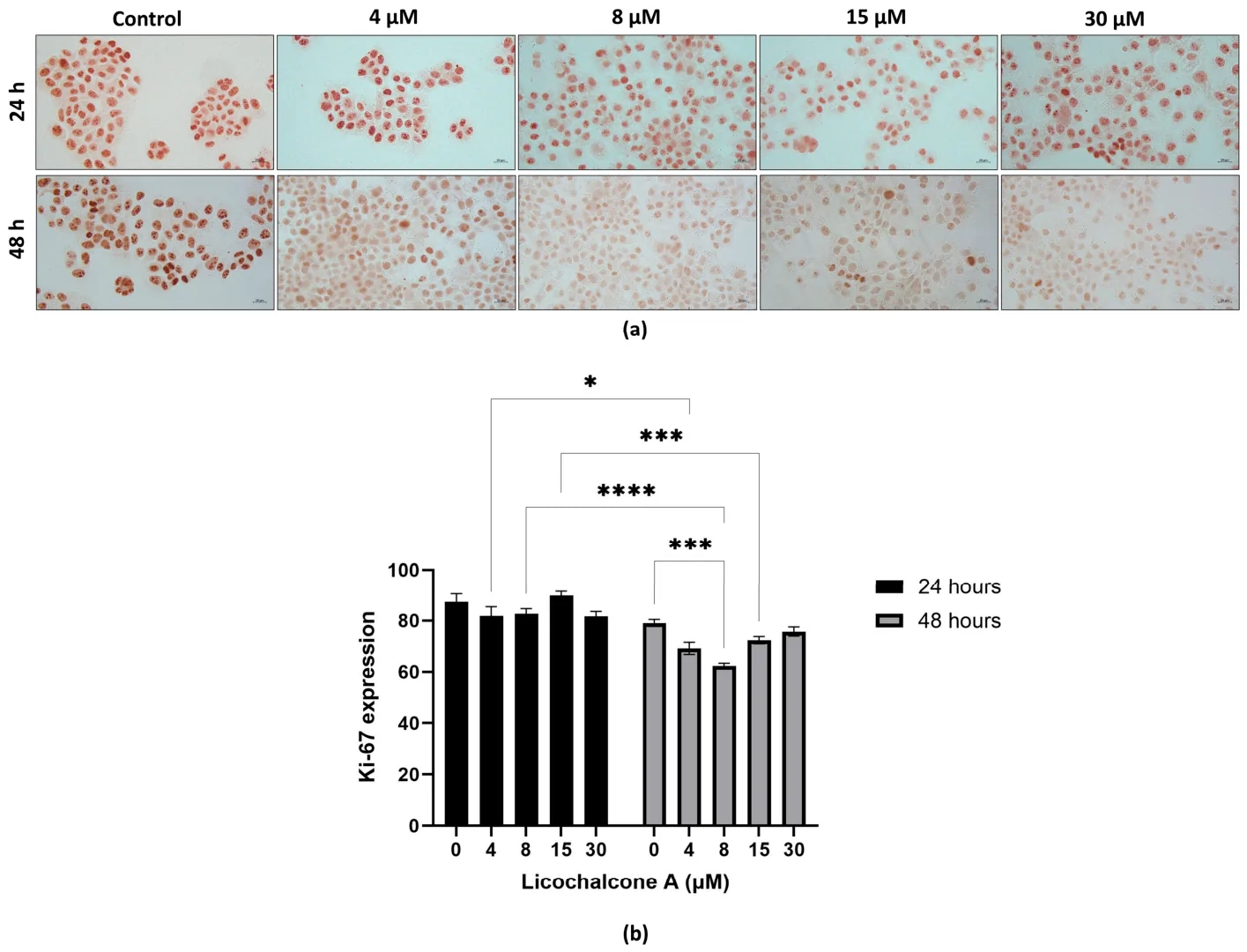
Effect of LA on Ki-67 expression in Ishikawa cells. (**a**) Representative immunocytochemical images of Ki-67 expression in Ishikawa cells following treatment with LA (0, 4, 8, 15, and 30 µM) for 24 and 48 h. (**b**) Semi-quantitative analysis of Ki-67 immunoreactivity expressed as the percentage of positively stained area (%Area). Scale bar = 20 µm. Data are presented as the mean ± SD from three independent experiments (n = 3). * *p* < 0.05, *** *p* < 0.001, and **** *p* < 0.0001.

The effect of LA on the proliferative activity of Ishikawa cells over time was evaluated by assessing Ki-67 immunoreactivity through immunocytochemistry following treatment with increasing concentrations of LA (0, 4, 8, 15, and 30 µM) for 24 and 48 h (Figure 5a). Quantitative analysis of the Ki-67-positive area (%Area) revealed time- and concentration-specific changes in Ki-67 expression (Figure 5b). At 24 h, LA treatment did not substantially change Ki-67 immunoreactivity compared with the 24 h untreated control. In contrast, prolonged LA exposure markedly reduced Ki-67 expression. Compared with the 24 h untreated control, Ki-67 expression was significantly reduced following 48 h of treatment with 4 µM (*p* < 0.0001), 8 µM (*p* < 0.0001), 15 µM (*p* = 0.0014), and 30 µM LA (*p* = 0.0228). Within the 48 h treatment groups, 8 µM LA produced the greatest reduction in Ki-67 expression and was significantly lower than the corresponding 48 h untreated control (*p* = 0.0003). However, increasing the LA concentration beyond 8 µM did not further reduce Ki-67 expression, indicating the absence of a consistent concentration-dependent response. Time-matched comparisons between 24 and 48 h further showed significant reductions in Ki-67 expression at 4 µM (*p* = 0.0102), 8 µM (*p* < 0.0001), and 15 µM (*p* = 0.0002).

### 2.6. Effects of LA on Migration and Invasion of Ishikawa Cells

**Figure 6:**
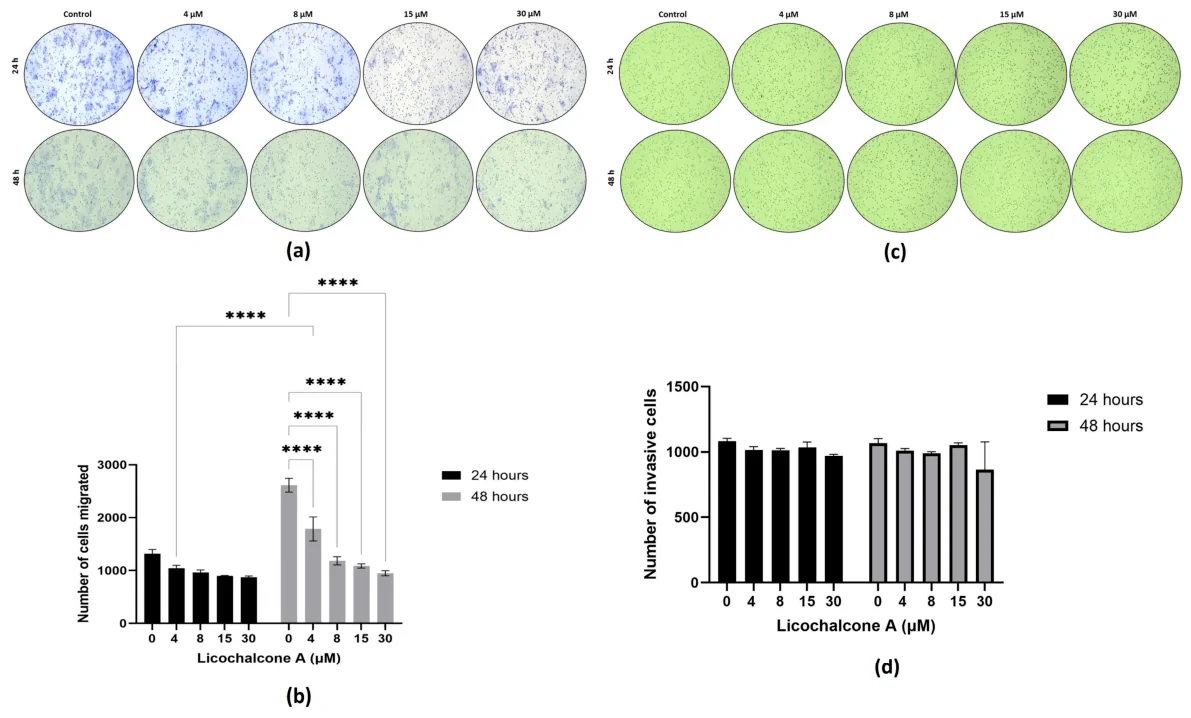
Effect of LA on the migration and invasion of Ishikawa cells. (**a**) Representative images of migrated Ishikawa cells following treatment with LA (0, 4, 8, 15, and 30 µM) for 24 and 48 h. (**b**) Quantitative analysis of the number of migrated cells. (**c**) Representative images of invasive Ishikawa cells following LA (0, 4, 8, 15, and 30 µM) treatment for 24 and 48 h. (**d**) Quantitative analysis of the number of invasive cells. Original magnification: ×400. Data are presented as the mean ± SD from three independent experiments (n = 3). **** *p* < 0.0001.

The effect of LA on the metastatic potential of Ishikawa endometrial cancer cells was evaluated using Transwell migration and invasion assays after 24 and 48 h treatments at various LA concentrations (0, 4, 8, 15, and 30 µM). In the migration assay, LA treatment did not significantly affect cell migration after 24 h compared with the corresponding untreated control. In contrast, after 48 h of treatment, LA markedly reduced the number of migrated cells at 4, 8, 15, and 30 µM compared with the 48 h untreated control (*p* < 0.0001 for all; Figure 6a,b). Furthermore, treatment duration differed significantly between the 24 and 48 h groups at 4 µM LA (*p* < 0.0001). These findings indicate that the effect of LA on Ishikawa cell migration was more pronounced following prolonged exposure. No statistically significant differences in the number of invasive cells were observed between the LA-treated groups and their corresponding untreated controls at any concentration tested (Figure 6c,d). Thus, under the current experimental conditions, LA treatment significantly reduced the number of migrated Ishikawa cells after 48 h, whereas no statistically significant change in the number of invaded cells was detected.

### 2.7. LA Modulates LC3-II Expression in Ishikawa Cells

**Figure 7:**
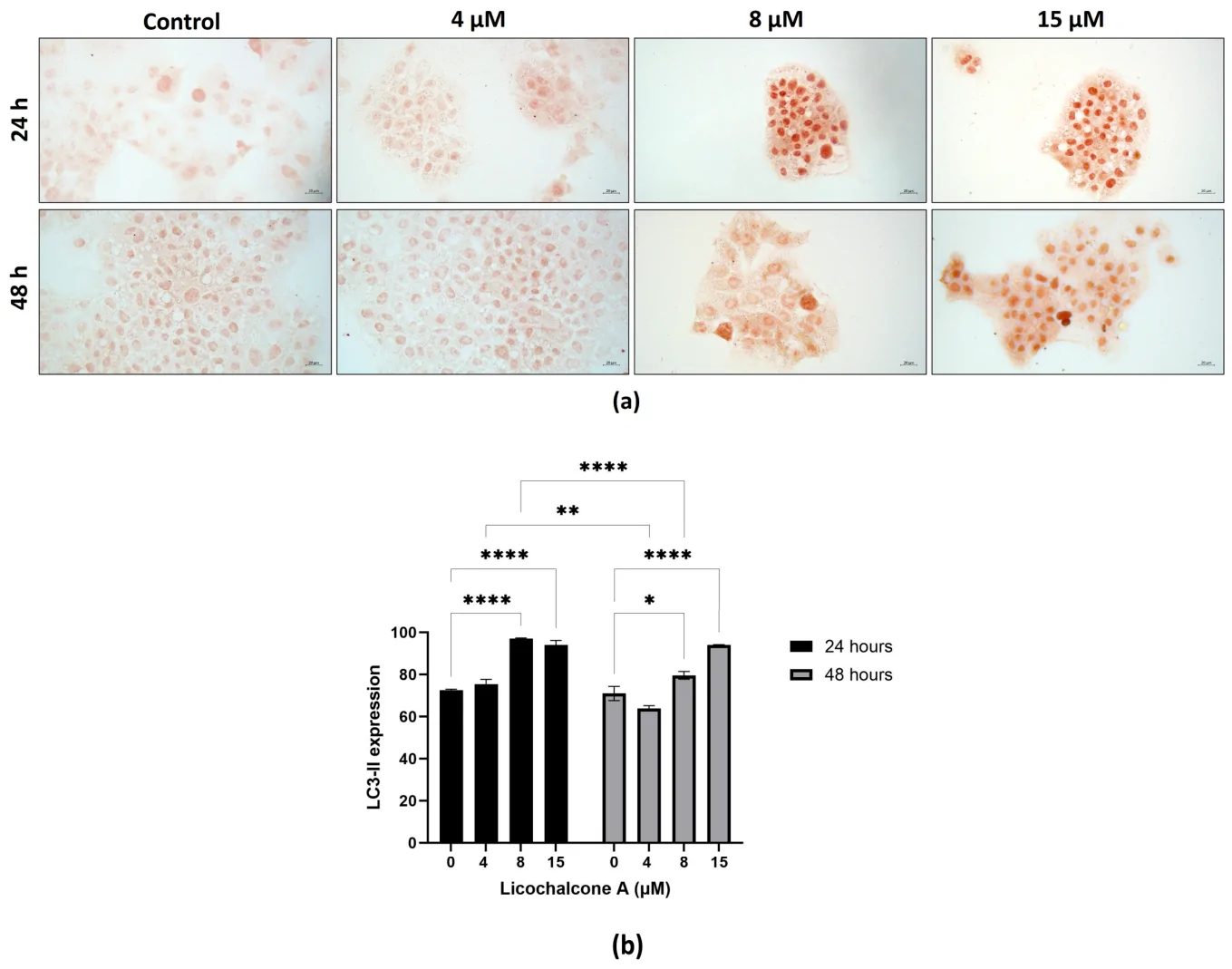
Effect of LA on LC3-II immunoreactivity in Ishikawa cells. (**a**) Representative immunocytochemical images of LC3-II expression following treatment with LA (0, 4, 8, and 15 µM) for 24 and 48 h. (**b**) Semi-quantitative analysis of LC3-II immunoreactivity expressed as the percentage of positively stained area (%Area). Scale bar = 20 µm. Data are presented as the mean ± SD from three independent experiments (n = 3). * *p* < 0.05, ** *p* < 0.01, and **** *p* < 0.0001.

To further characterize LC3-related molecular responses to LA, LC3-II immunoreactivity was evaluated by immunocytochemistry following treatment with LA (0, 4, 8, and 15 µM) for 24 and 48 h (Figure 7a). Semi-quantitative analysis of the LC3-II-positive area (%Area) revealed concentration and time-dependent changes in LC3-II immunoreactivity (Figure 7b).

At 24 h, LC3-II immunoreactivity increased significantly after treatment with 8 and 15 µM LA compared with the corresponding untreated control (*p* < 0.0001 for both), whereas 4 µM showed no significant difference. At 48 h, LC3-II immunoreactivity was significantly increased at 8 µM (*p* = 0.0391) and 15 µM (*p* < 0.0001) compared with the corresponding untreated control.

Time-matched comparisons between 24 and 48 h further showed significant reductions in LC3-II immunoreactivity at 4 µM (*p* = 0.0166) and 8 µM (*p* = 0.0002), whereas no significant time-dependent change was observed at 15 µM.

### 2.8. LA Alters PI3K, Akt1, mTOR, and MAP1LC3B mRNA Expression in Ishikawa Cells

**Figure 8:**
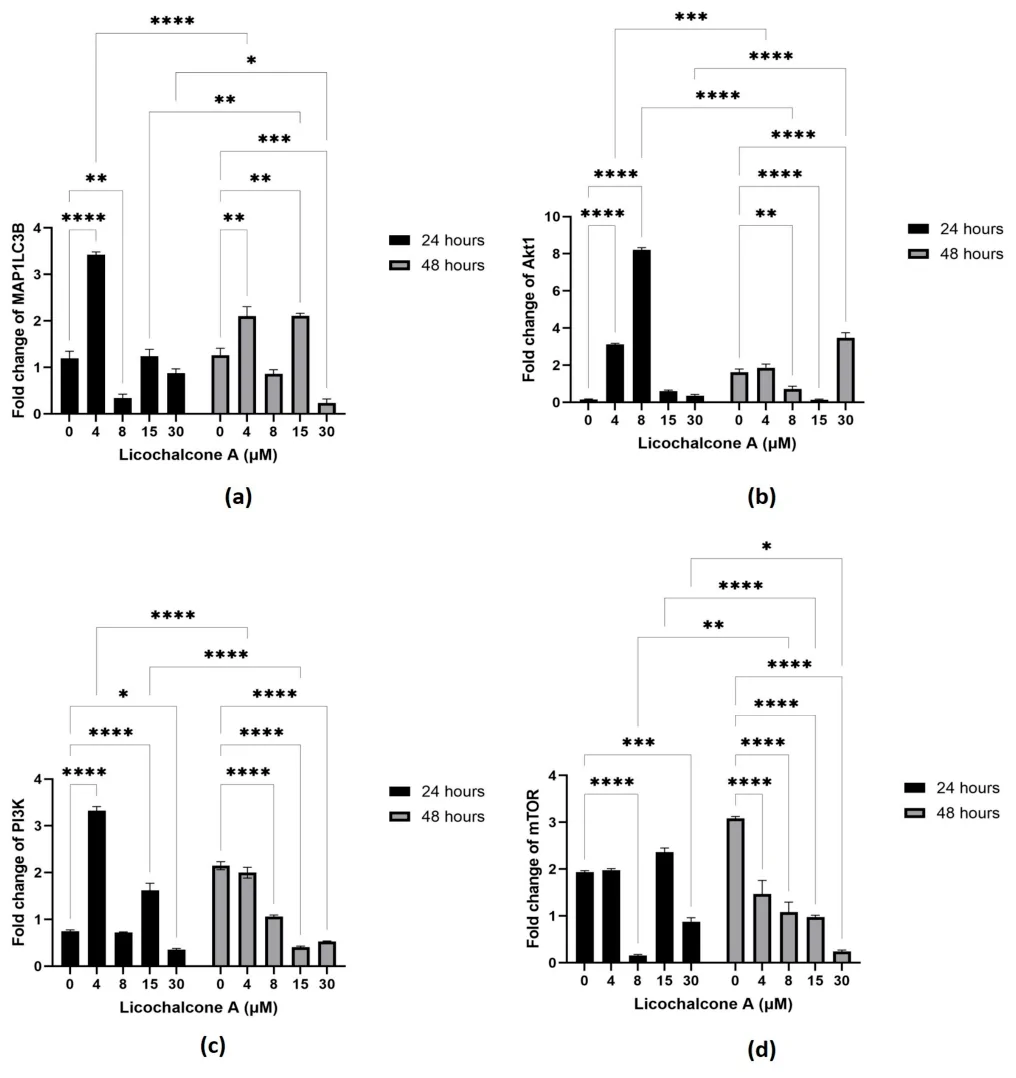
Effect of LA on MAP1LC3B, Akt1, PI3K, and mTOR mRNA expression in Ishikawa cells. (**a**) Relative mRNA expression levels of MAP1LC3B, (**b**) Akt1, (**c**) PI3K, and (**d**) mTOR following treatment with LA (0, 4, 8, 15, and 30 µM) for 24 and 48 h were determined by RT-qPCR. Relative gene expression was calculated using the 2^−ΔΔCt^ method, with β-actin as the reference gene and the corresponding untreated control at each time point serving as the calibrator. Data are presented as mean ± SD. * *p* < 0.05, ** *p* < 0.01, *** *p* < 0.001, and **** *p* < 0.0001.

RT-qPCR analysis was performed to investigate the effects of LA on the expression of the autophagy-related marker MAP1LC3B and the PI3K/Akt/mTOR pathway components in Ishikawa cells following treatment with 0, 4, 8, 15, and 30 µM LA for 24 and 48 h. Overall, LA induced distinct concentration- and time-dependent changes in MAP1LC3B, Akt1, PI3K, and mTOR mRNA expression (Figure 8).

MAP1LC3B expression exhibited a similarly heterogeneous response. At 24 h, MAP1LC3B mRNA expression was significantly increased at 4 µM (*p* < 0.0001) but significantly reduced at 8 µM (*p* = 0.0024) compared with the untreated control. No significant differences were detected at 15 or 30 µM. At 48 h, MAP1LC3B expression increased significantly at 4 and 15 µM (*p* = 0.0029 and *p* = 0.0027, respectively), whereas it decreased significantly at 30 µM (*p* = 0.0002); the change at 8 µM was not significant. Comparing 24 and 48 h, MAP1LC3B expression decreased significantly at 4 µM (*p* < 0.0001), increased significantly at 15 µM (*p* = 0.0020), and decreased significantly at 30 µM (*p* = 0.0323). In contrast, no significant temporal change was detected at 8 µM (Figure 8a).

Akt1 expression also showed a concentration- and time-dependent response. At 24 h, Akt1 mRNA levels increased significantly at 4 and 8 µM compared with the untreated control (*p* < 0.0001 for both), whereas the changes at 15 and 30 µM were not statistically significant. At 48 h, Akt1 expression was significantly reduced at 8 µM (*p* = 0.0075) and 15 µM (*p* < 0.0001), but was significantly increased at 30 µM (*p* < 0.0001); no significant difference was observed at 4 µM. Comparing treatment durations, Akt1 expression decreased significantly from 24 to 48 h at 4 µM (*p* = 0.0002) and 8 µM (*p* < 0.0001), whereas expression increased significantly over time at 30 µM (*p* < 0.0001). No significant temporal difference was observed at 15 µM (Figure 8b). At 24 h, PI3K expression was significantly increased at 4 µM (*p* < 0.0001) and 15 µM (*p* < 0.0001) compared with the untreated control, whereas a significant reduction was observed at 30 µM (*p* = 0.0336). No significant difference was detected at 8 µM. After 48 h, PI3K expression was significantly reduced at 8, 15, and 30 µM (*p* < 0.0001 for all), while the change at 4 µM was not significant. Time-dependent comparisons further showed significant reductions from 24 to 48 h at 4 and 15 µM (*p* < 0.0001 for both), whereas no significant temporal change was detected at 8 or 30 µM (Figure 8c).

For mTOR, no significant alteration was detected at 4 or 15 µM after 24 h, whereas mTOR expression was significantly reduced at 8 µM (*p* < 0.0001) and 30 µM (*p* = 0.0002). After 48 h, mTOR expression was significantly reduced at all tested LA concentrations compared with the corresponding untreated control (*p* < 0.0001 for 4, 8, 15, and 30 µM). Time-dependent analysis showed a significant increase in mTOR expression from 24 to 48 h at 8 µM (*p* = 0.0011), whereas 15 µM (*p* < 0.0001) and 30 µM (*p* = 0.0439) showed significant decreases. No significant temporal difference was observed at 4 µM (Figure 8d). Collectively, these findings indicate that LA modulates MAP1LC3B, Akt1, PI3K, and mTOR transcription in a concentration and time-dependent manner, with variable responses among pathway components. Notably, prolonged LA exposure consistently suppressed mTOR expression across all tested concentrations, whereas MAP1LC3B, Akt1, and PI3K exhibited more variable responses depending on treatment concentration and duration.

### 2.9. Licochalcone A Modulates Akt Phosphorylation in a Time and Concentration-Specific Manner

**Figure 9:**
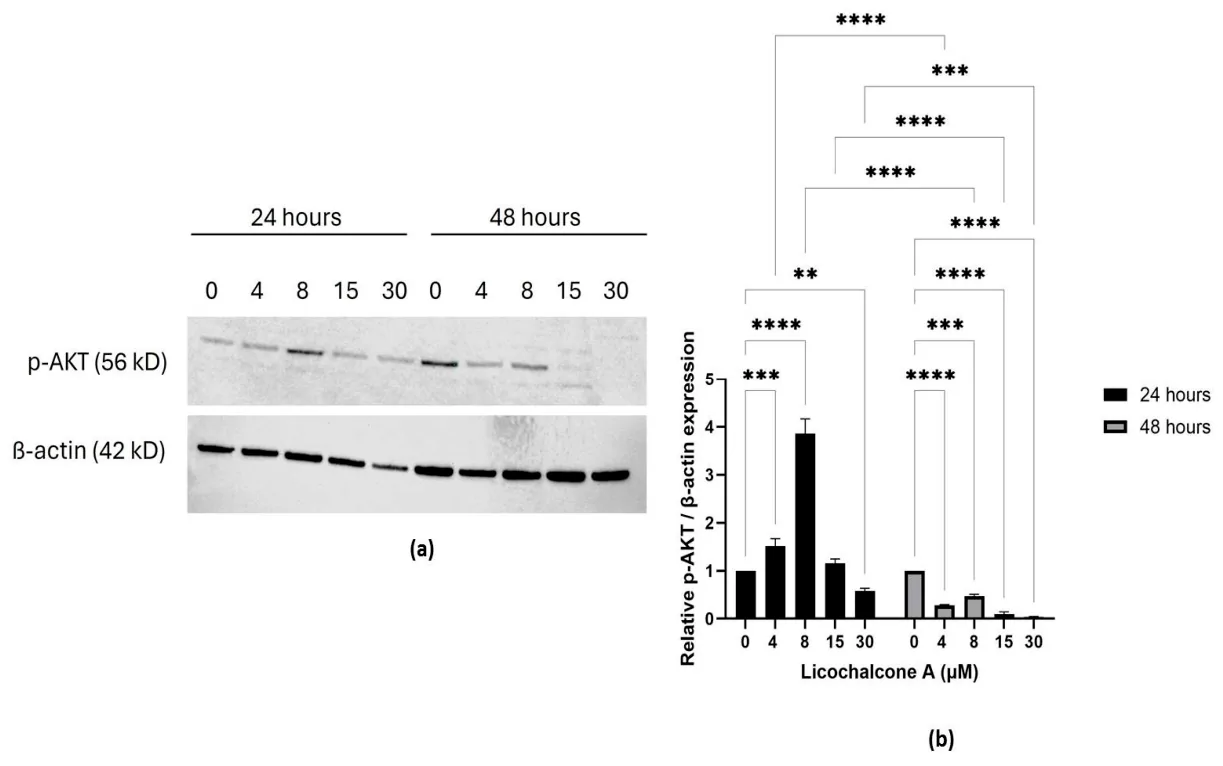
Effect of LA on Akt phosphorylation in Ishikawa cells. (**a**) Representative Western blot bands showing phosphorylated Akt (p-Akt) and β-actin expression following treatment with LA (0, 4, 8, 15, and 30 µM) for 24 and 48 h. (**b**) Densitometric analysis of p-Akt expression normalized to β-actin. Data are presented as mean ± SD from three independent experiments (n = 3). ** *p* < 0.01, *** *p* < 0.001, and **** *p* < 0.0001.

Western blot analysis was performed to evaluate the effect of LA on Akt phosphorylation in Ishikawa cells following treatment with 0, 4, 8, 15, and 30 µM LA for 24 and 48 h (Figure 9).

At 24 h, LA induced a concentration-dependent but nonlinear modulation of Akt phosphorylation. Compared with the untreated control, p-Akt expression was significantly increased at 4 µM (*p* = 0.0007) and 8 µM (*p* < 0.0001), whereas no significant change was observed at 15 µM. In contrast, treatment with 30 µM LA significantly reduced p-Akt expression (*p* = 0.0076; Figure 9b). After 48 h, the response differed markedly. p-Akt levels normalized to β-actin were significantly reduced at all tested LA concentrations compared with the corresponding untreated control, with significant decreases at 4 µM (*p* < 0.0001), 8 µM (*p* < 0.001), 15 µM (*p* < 0.0001), and 30 µM (*p* < 0.0001; Figure 9b).

Direct comparison of the two treatment durations further demonstrated a significant time-dependent reduction in Akt phosphorylation at each LA concentration. p-Akt expression was significantly lower at 48 h than at 24 h following treatment with 4 µM (*p* < 0.0001), 8 µM (*p* < 0.0001), 15 µM (*p* < 0.0001), and 30 µM LA (*p* = 0.0003; Figure 9b). Overall, these findings show a clear time-dependent shift in the Akt phosphorylation response to LA, with higher p-Akt levels at lower concentrations during early treatment, followed by pronounced reductions across all tested concentrations after prolonged treatment.

## 3. Discussion

Natural products and their bioactive constituents continue to attract considerable interest in anticancer drug discovery because of their compositional diversity and ability to act on multiple cellular targets and signaling pathways [26]. Among these compounds, LA, a bioactive chalcone derived from Glycyrrhiza species, has emerged as a promising candidate because of its anti-inflammatory, antioxidant, and antitumor properties [9,10,27]. Accumulating evidence indicates that LA can interfere with several hallmarks of malignant behavior, including sustained proliferation, resistance to cell death, cell cycle progression, migration, and invasion [9,28]. However, its effects in Ishikawa endometrial cancer cells and the associated molecular responses remain incompletely understood. In the present study, LA exerted antiproliferative, cytotoxic, and antimigratory effects in Ishikawa endometrial cancer cells, as shown by reduced metabolic activity and clonogenic capacity, decreased Ki-67 expression after prolonged exposure, increased apoptosis, S-phase accumulation, and suppressed cell migration. These functional effects were accompanied by concentration and time-dependent changes in PI3K/Akt/mTOR- and LC3-II-related molecular responses, with prolonged LA exposure particularly associated with reduced Akt phosphorylation and mTOR transcript levels.

This study showed that LA suppresses the growth of Ishikawa endometrial cancer cells through multiple cellular effects, including metabolic activity, clonogenic capacity, proliferative activity, cell cycle progression, and apoptosis. LA reduced metabolic activity in a concentration-dependent manner and markedly impaired colony formation, with the inhibitory effect becoming more pronounced at higher concentrations and with prolonged exposure. These findings are consistent with previous studies showing that LA inhibits proliferation and colony formation in osteosarcoma cells [23], reduces cell viability in gastric cancer cells [24], and suppresses cell viability and colony formation in pancreatic ductal adenocarcinoma [29]. Concentration-dependent inhibition of proliferation has also been reported in U87 MG and U251 glioma cells [30]. Collectively, these observations indicate that the antiproliferative activity previously attributed to LA in other cancer models also extends to Ishikawa endometrial cancer cells.

The reduction in proliferative capacity was further supported by Ki-67 immunocytochemistry and cell cycle analysis. Ki-67 is closely associated with active cell cycle progression and is widely used to estimate the proliferative fraction of tumor cells [31]. Although Ki-67 expression was not significantly altered after 24 h of LA treatment, a marked reduction was observed after 48 h, indicating that suppression of proliferative activity becomes more evident with prolonged exposure. Notably, Ki-67 expression decreased most at 8 µM, with no further reduction at higher concentrations, indicating no consistent concentration-dependent response. Although the mechanism underlying this pattern cannot be determined from the present data, it may reflect concentration-specific differences in the cellular response to LA. Importantly, the time-dependent reduction in Ki-67 expression parallels the stronger suppression of clonogenic capacity observed after prolonged treatment, supporting a sustained inhibitory effect of LA on proliferative potential. Previous findings are consistent with this interpretation, as LA reduced Ki-67-positive tumor cells in glioma xenografts [30] and decreased Ki-67 expression in esophageal squamous cell carcinoma xenografts [32]. Similarly, LA reduced the proportion of EdU-positive proliferating cells in pancreatic ductal adenocarcinoma cells [29], further supporting its inhibitory effect on tumor cell proliferation.

Cell cycle analysis provided further insight into the mechanisms underlying this growth inhibition. LA altered cell cycle distribution, with the most prominent changes observed at 15 and 30 µM, where the G0/G1 population decreased and cells accumulated in S phase without a substantial change in the G2/M population. This pattern suggests interference with normal cell cycle progression and may contribute to the reduced proliferative and clonogenic capacity observed following LA treatment. The specific phase affected by LA appears to vary across tumor models: G2/M arrest has been reported in esophageal cancer cells [7], whereas S-phase arrest has been described in HeLa cells and combined S and G2/M-phase arrest in SCC-25 cells [33]. Suppression of cell cycle progression has also been reported in osteosarcoma and pancreatic cancer models [23,29]. Thus, although the precise cell cycle response may vary by cancer cell type and experimental conditions, disruption of cell cycle progression appears to be a recurring component of LA’s antiproliferative activity.

Increased apoptosis represented another aspect of the cellular response to LA. Resistance to cell death is a fundamental feature of malignant cells and contributes to tumor progression and therapeutic resistance [34,35]. In the present study, Annexin V/7-AAD analysis demonstrated a significant increase in total apoptosis at 15 and 30 µM LA. Although the overall proportion of apoptotic cells remained relatively modest, the significant increase observed at higher concentrations suggests that apoptosis contributes to LA-induced growth inhibition. This observation is consistent with the previous studies demonstrating LA-induced apoptosis in HepG2 hepatocellular carcinoma cells [10], A549 and H460 lung cancer cells [36], esophageal cancer cells [7], and glioma cells [30]. In vivo, LA treatment has also been associated with increased TUNEL positivity in glioma xenografts [30] and increased cleaved caspase-3 expression in esophageal squamous cell carcinoma xenografts [32]. Overall, these observations support the view that reduced Ishikawa cell growth is associated with multiple cellular responses, including impaired proliferation, altered cell cycle progression, and increased apoptosis.

Beyond these effects on cell growth and survival, LA also influenced Ishikawa cell migratory behavior. Although LA treatment did not significantly alter migration after 24 h, migration was markedly reduced at all tested concentrations after 48 h compared with the corresponding untreated control, indicating that this effect became more evident with prolonged exposure. Similar antimigratory effects of LA have been reported in other cancer models. Hao et al. demonstrated concentration-dependent inhibition of migration and invasion in oral squamous cell carcinoma cells, associated with PI3K/Akt pathway modulation [37]. Similarly, Yu et al. showed that LA inhibited both migration and invasion in glioma cells [30], while Peng et al. reported suppression of migration accompanied by changes in migration-associated markers in PDAC cells [29]. Collectively, these findings suggest that suppression of tumor cell migration is a recurring feature of LA’s anticancer activity across different cancer models.

In contrast, LA did not significantly affect the invasive capacity of Ishikawa cells under the experimental conditions used in the present study. Although migration and invasion are closely related processes involved in metastatic progression, invasion additionally requires interactions with and degradation of extracellular matrix barriers and therefore involves a broader range of cellular processes. Therefore, the marked suppression of migration in the absence of a significant change in invasion suggests that LA may differentially affect these two processes in Ishikawa cells. Moreover, serum-free medium was used in the upper chamber of the Transwell assays to minimize the potential contribution of cell proliferation, supporting the interpretation that the observed reduction in migration cannot be attributed solely to suppression of proliferative activity. Nevertheless, given the concurrent effects of LA on metabolic activity, proliferation, and apoptosis, the present findings are insufficient to establish a direct effect of LA on cell motility. Thus, the absence of significant invasion inhibition in Ishikawa cells, despite the inhibitory effects reported in oral squamous cell carcinoma and glioma models [30,37], may reflect tumor-specific biological differences or differences in experimental conditions. Further investigation of epithelial–mesenchymal transition markers, matrix metalloproteinases, and proteins involved in extracellular matrix remodeling may help clarify the molecular basis underlying the differential effects of LA on migration and invasion.

The PI3K/Akt/mTOR pathway is a major signaling network involved in the regulation of cell proliferation, survival, metabolism, migration, and resistance to apoptosis, and is frequently dysregulated in human cancers [11,12,16]. Aberrant activation of this pathway is also well established in endometrial cancer, where it has been associated with tumor progression and resistance to therapy-induced apoptosis [13,14,15]. Consistent with its role in cell survival, pharmacological inhibition of Akt has been shown to promote apoptosis in ovarian and endometrial cancer cells [13].

Previous studies have also linked the anticancer activity of LA to modulation of PI3K/Akt/mTOR signaling. Hao et al. showed that LA inhibited proliferation, migration, and invasion in SCC4 and CAL-27 oral squamous cell carcinoma cells, associated with modulation of PI3K/Akt signaling [37]. In breast cancer cells, suppression of PI3K/Akt/mTOR pathway activation by LA was accompanied by increased autophagy [20], while in cervical cancer cells, inactivation of this pathway was implicated in LA-induced autophagy [21]. PI3K/Akt and mTOR signaling have also been identified among the major molecular pathways associated with the anticancer effects of LA in reviews of the available experimental evidence [28,33]. Consistent with these findings, and given the cellular effects of LA observed in the present study, we evaluated changes in PI3K/Akt/mTOR pathway components to further characterize the molecular response of Ishikawa cells to LA.

RT-qPCR analysis revealed distinct transcriptional responses among PI3K/Akt/mTOR pathway components following LA treatment. PI3K and Akt1 expression varied with LA concentration and treatment duration, indicating that the pathway’s upstream components were not uniformly altered at the transcriptional level. In contrast, mTOR showed a more consistent reduction with prolonged exposure. At 24 h, mTOR transcript levels were significantly decreased at 8 and 30 µM, whereas after 48 h, significant reductions were observed at all tested LA concentrations. Thus, despite the variable transcriptional responses of PI3K and Akt1, prolonged LA exposure was associated with a consistent reduction in mTOR transcript levels. Given the central role of mTOR as a downstream component of PI3K/Akt signaling and a key regulator of cellular growth, its consistent reduction after 48 h represents a notable feature of the molecular response to prolonged LA exposure.

The analysis of Akt phosphorylation further demonstrated that the molecular response to LA varied with concentration and exposure duration. At 24 h, β-actin-normalized p-Akt levels increased at 4 and 8 µM, remained unchanged at 15 µM, and decreased at 30 µM. In contrast, after 48 h, p-Akt levels were markedly reduced at all tested LA concentrations. The concurrent reduction in β-actin-normalized p-Akt levels and mTOR transcript levels after 48 h suggests that prolonged LA exposure is associated with changes in Akt/mTOR-related signaling in Ishikawa cells. However, because total Akt, PI3K activity, and total and phosphorylated mTOR were not evaluated, these findings should not be interpreted as direct evidence of PI3K/Akt/mTOR pathway inhibition. Further evaluation of these pathway components is required to more precisely define the involvement of PI3K/Akt/mTOR signaling in the molecular response of Ishikawa cells to LA.

Given the close regulatory relationship between PI3K/Akt/mTOR signaling and autophagy, we also evaluated MAP1LC3B transcript levels and LC3-II immunoreactivity to characterize autophagy-related molecular changes in LA-treated Ishikawa cells. MAP1LC3B transcript levels showed a variable response according to LA concentration and exposure duration, with increases under some treatment conditions and decreases under others, indicating the absence of a consistent directional change at the transcriptional level. LC3-II immunoreactivity was significantly increased at 8 and 15 µM compared with the corresponding controls at both 24 and 48 h. However, comparisons between treatment durations revealed significant reductions in LC3-II immunoreactivity from 24 to 48 h at 4 and 8 µM, whereas no significant time-dependent change was observed at 15 µM.

Previous studies have also linked LA to autophagy-related responses in different cancer models. Xue et al. showed that LA promoted autophagy in breast cancer cells alongside inhibition of PI3K/Akt/mTOR signaling [20], while Tsai et al. reported a similar relationship in cervical cancer cells [21]. In LNCaP prostate cancer cells, LA increased LC3 processing and induced autophagic vacuole formation while reducing mTOR phosphorylation, whereas p-Akt remained largely unchanged [38]. Peng et al. likewise reported increased LC3-II and p62 expression in LA-treated MIA PaCa-2 pancreatic cancer cells, but not in BxPC3 cells [28], indicating that autophagy-related responses to LA may differ between cancer cell types. Evidence from a non-cancer model further supports an association between LA and autophagy-related signaling, with LA-mediated mTOR inhibition accompanied by regulation of the ULK1/Beclin1/VPS34 pathway and increased LC3B expression [39]. Collectively, these studies demonstrate that LA can modulate autophagy-related markers and processes, although the nature of the response may vary depending on the cellular model and experimental conditions. The concentration- and time-dependent changes in MAP1LC3B transcript levels and LC3-II immunoreactivity observed in Ishikawa cells are broadly consistent with these findings. However, because autophagic flux was not directly assessed in the present study, the functional significance of these changes requires further investigation using complementary autophagic flux analyses.

Several limitations of this study warrant consideration. First, the experiments were conducted in a single endometrial cancer cell line; therefore, validation in additional endometrial cancer models with different molecular backgrounds is warranted. Second, although we assessed Akt phosphorylation at the protein level, we did not simultaneously evaluate total Akt, nor did we comprehensively assess other components of the PI3K/Akt/mTOR pathway at the protein or phosphorylation level. Assessment of the p-Akt/total Akt ratio, PI3K protein/activity, and total and phosphorylated mTOR would provide a more complete evaluation of pathway status and help clarify the molecular involvement of PI3K/Akt/mTOR signaling in the response to LA. Third, although changes in MAP1LC3B expression and LC3-II immunoreactivity indicate alterations in autophagy-related markers, we did not directly assess autophagic flux. Complementary analyses, including autophagic flux assays and additional markers, would be required to determine the functional significance of these changes. Fourth, although serum-free medium was used in the upper chamber of the Transwell assays to minimize the contribution of cell proliferation, LA also reduced proliferative capacity and metabolic activity and increased apoptosis. Therefore, the extent to which the observed reduction in migration reflects a direct antimigratory effect rather than a secondary consequence of these cellular effects cannot be fully distinguished. Future studies using proliferation-controlled migration assays or appropriate normalization strategies will be required to clarify this issue. Finally, the associations between the molecular changes in Akt/mTOR-related signaling and the cellular effects of LA remain correlative. Pathway-specific inhibition experiments would help determine whether these molecular changes are causally involved in the antiproliferative, cytotoxic, and antimigratory effects observed in Ishikawa cells.

## 4. Materials and Methods

### 4.1. Cell Culture

The Ishikawa human endometrial carcinoma cell line used in this study was originally obtained from the American Type Culture Collection (ATCC; CRL-12051; Manassas, VA, USA) and was kindly provided to us by Dr. Meral Koyutürk (Istanbul University-Cerrahpasa, Faculty of Medicine, Istanbul, Türkiye). Mycoplasma-free cells were maintained as a monolayer culture in the Cell Culture Laboratory of the Department of Histology and Embryology, Istanbul Faculty of Medicine. Cells were cultured in Dulbecco’s Modified Eagle Medium (DMEM; DMEM-12-A, Capricorn Scientific, Ebsdorfergrund, Germany) supplemented with 10% fetal bovine serum (FBS; FBS-11A, Capricorn Scientific, Ebsdorfergrund, Germany), 1% antibiotic/antimycotic (Cat. No. 15240062, Gibco, Grand Island, NY, USA) at 37 °C in a humidified atmosphere containing 5% CO_2_. Cells were passaged upon attaining approximately 70–80% confluence using 0.05% trypsin-EDTA (Cat. No. 27250018, Gibco, Grand Island, NY, USA). Cell viability was assessed by trypan blue exclusion, and cells with viability above 90% were used for subsequent experiments.

### 4.2. MTT Assay

Cells were treated with increasing concentrations of LA (Cat. No. 68783, Sigma-Aldrich, St. Louis, MO, USA) at 0, 1, 3, 5, 10, 20, 30, and 40 μM for 24 and 48 h, while untreated cells served as the control group. Dimethyl sulfoxide (DMSO; Cat. No. D8418; Sigma-Aldrich, St. Louis, MO, USA) was used as solvent for LA. The final concentration of DMSO did not exceed 0.1% (*v*/*v*), a concentration that did not affect cell viability. Following treatment, the MTT assay was performed according to the manufacturer’s instructions using an MTT assay kit (E-CK-A341, Elabscience, Houston, TX, USA). Absorbance was measured at 570 nm using a microplate reader (AMR-100, Cat. No. AS-16050-00, Hangzhou Allsheng Instruments Co., Ltd., Hangzhou, China). The IC_50_ value was determined from the 24 h dose–response analysis and used to select the LA concentrations for the subsequent 24 and 48 h experiments. All experiments were performed in three independent biological replicates.

### 4.3. Colony Formation Assay

A colony formation assay was performed to evaluate the long-term proliferative capacity of Ishikawa cells following LA treatment. Cells were cultured in 6-well plates at a density of 1 × 10^3^ cells/well and exposed to LA at concentrations of 0, 4, 8, 15, and 30 µM for 24 or 48 h. Following the treatment period, the LA-containing medium was removed and replaced with LA-free culture medium. Cells were then maintained under standard culture conditions for a total colony formation period of 14 days. At the end of the 14 day period cells were fixed with methanol (ACS reagent, ≥99.8%; Cat. No. 320390; Sigma-Aldrich, St. Louis, MO, USA) for 15 min and stained with 0.25% crystal violet (Cat. No. C0775; Sigma-Aldrich, St. Louis, MO, USA) solution for 10 min. Colonies containing more than 50 cells were considered clonogenic colonies and were quantified using ImageJ software(ImageJ-FiJi, version 1.54f; National Institutes of Health, Bethesda, MD, USA), as previously described [40,41]. All experiments were performed in three independent biological replicates.

### 4.4. Flow Cytometric Analysis

Flow cytometric analyses quantitatively evaluated cell cycle progression and apoptosis in Ishikawa cells following LA treatment. Cells were seeded at a density of 1 × 10^5^ cells per group and treated with the selected effective LA concentrations for 24 h at 37 °C in a humidified atmosphere containing 5% CO_2_. Following treatment, cells were detached using trypsin/EDTA, collected by centrifugation, and washed three times with phosphate-buffered saline (PBS; Cat. No. P4417; Sigma-Aldrich, St. Louis, MO, USA). Cell cycle distribution was analyzed using a Cell Cycle Assay Kit (E-CK-A352, Elabscience, Houston, TX, USA), while apoptosis was assessed using an Annexin V-FITC Apoptosis Staining/Detection Kit (Cat. No. 640922, BioLegend, San Diego, CA, USA), according to the manufacturers’ instructions. For quantitative analysis, total apoptosis was calculated as the sum of early and late apoptotic cell populations; necrotic cells were excluded from the total apoptosis calculation. Samples were then analyzed by BD Accuri™ C6 flow cytometer (BD Biosciences, San Jose, CA, USA). All analyses were performed according to the respective kit protocols.

### 4.5. Immunocytochemical Analysis

Immunocytochemical (ICC) analysis was performed to evaluate LA’s effects on cell proliferation and autophagy in Ishikawa cells. Ki-67 expression was assessed as a marker of cellular proliferation, while LC3-II immunoreactivity was evaluated to characterize LC3-related cellular responses. Cells were seeded onto sterile round coverslips and treated with the selected concentrations of LA. Following treatment, cells were fixed with 4% paraformaldehyde (powder, 95%; Cat. No. 158127; Sigma-Aldrich, St. Louis, MO, USA) and permeabilized with 0.2% Triton X-100 (Cat. No. T8787; Sigma-Aldrich, St. Louis, MO, USA) in PBS. After blocking with 1% bovine serum albumin (BSA; Cat. No. A9647; Sigma-Aldrich, St. Louis, MO, USA), cells were incubated with the respective primary antibodies for 1 h at 37 °C, then incubated with a horseradish peroxidase-conjugated secondary antibody and streptavidin–peroxidase (SHP125, ScyTek Laboratories, Logan, UT, USA). Immunoreactivity was visualized using 3-amino-9-ethylcarbazole (AEC) as the chromogen (Cat. No. TA-125-HA, Thermo Scientific, Waltham, MA, USA). Samples were examined under a light microscope, and Ki-67 (orb227859, Biorbyt, Cambridge, UK, used at 1:200 dilution) and LC3-II (sc-271625, Santa Cruz Biotechnology, Santa Cruz, CA, USA, used at 1:100 dilution) immunoreactivity was semi-quantitatively assessed using ImageJ software. A consistent intensity threshold was applied to all images acquired under identical imaging parameters, and the percentage of positively stained area (%Area) was calculated for each image. The resulting %Area values were used for statistical comparisons among the experimental groups [42].

### 4.6. Transwell Migration and Invasion Assays

The effects of LA on the migratory and invasive capacities of Ishikawa cells were evaluated using Transwell assays. Briefly, 24-well Transwell chambers with 6.5 mm polycarbonate membranes containing 8 μm pores (Cat. No. 3422, Corning, Corning, NY, USA) were used. For the invasion assay, the upper surface of the membranes was coated with Matrigel (Cat. No. 356234, Corning, Corning, NY, USA, used at 50 μL/cm^2^) and incubated at 37 °C for 1 h to form a gel. In contrast, uncoated membranes were used for the migration assay. Ishikawa cells (2 × 10^6^ cells/well) suspended in serum-free medium containing the selected concentrations of LA were seeded into the upper chambers. Using serum-free medium in the upper chamber was intended to minimize the potential contribution of cell proliferation to the assay. In contrast, 700 μL of culture medium containing 10% FBS was added to the lower chambers as a chemoattractant. The chambers were incubated for 24 or 48 h at 37 °C in a humidified atmosphere containing 5% CO_2_. Following the respective incubation periods, non-migrated or non-invaded cells were gently removed from the upper surface of the membranes with a cotton swab. Cells that had migrated or invaded to the lower surface of the membranes were fixed with 70% ethanol (Cat. No. 1.00983; Supelco, Merck KGaA, Darmstadt, Germany) for 20 min and stained with 0.4% crystal violet. All experiments were performed in three independent biological replicates. Cells were visualized using an inverted microscope, and five randomly selected fields from each membrane were photographed and analyzed.

### 4.7. Reverse Transcription Quantitative Real-Time PCR (RT-qPCR)

RT-qPCR analysis was performed to evaluate the effects of LA on the PI3K/Akt/mTOR- and LC3-related transcript levels in Ishikawa cells. Total RNA was extracted from the cells using a commercial RNA purification kit according to the manufacturer’s instructions (A.B.T.™ Blood/Tissue RNA Purification Kit—Atlas Biotechnology, Ankara, Türkiye, I04-01-10) RNA concentration and purity were determined spectrophotometrically. Subsequently, complementary DNA (cDNA) was synthesized from the isolated RNA using a commercial reverse transcription kit (VitaScript™ FirstStrand cDNA Synthesis Kit—Procomcure Biotech GmbH, Bergheim, Austria, PCCSKU1301) Quantitative PCR was performed using gene-specific primers for MAP1LC3B, PI3K, AKT1, and mTOR and a commercial qPCR kit (2X Magic SYBR Mix—Procomcure Biotech GmbH, Bergheim Austria, PCCSKU1107) (Table 1). The amplification protocol consisted of an initial denaturation at 95 °C for 5 min, followed by 40 cycles of 95 °C for 15 s, 60 °C for 30 s, and 72 °C for 30 s (Applied Biosystem 7500 Real Time PCR System, Foster City, CA, USA). Following amplification, melt curve analysis was performed from 60 °C to 95 °C with fluorescence acquisition at 0.2 °C increments to assess the amplification products. Relative gene expression was calculated using the 2^−ΔΔCt method, with β-actin as the reference gene and the untreated control at each time point as the calibrator. Three independent biological replicates (n = 3) were analyzed for each treatment condition.

### 4.8. Western Blot Analysis

Western blot analysis was performed to evaluate the effect of LA on Akt signaling in Ishikawa cells. Following treatment, cells were collected and lysed in radioimmunoprecipitation assay (RIPA) buffer (Cat. No. R0278; Sigma-Aldrich, St. Louis, MO, USA) supplemented with a protease inhibitor cocktail (Cat. No. 78429; Thermo Scientific, Rockford, IL, USA). Protein concentrations were determined, and equal amounts of protein were separated by SDS-PAGE and transferred onto membranes. After blocking with 5% non-fat dry milk prepared in Tris-buffered saline containing 0.1% Tween-20 (T-TBS; (Cat. No. P1379; Sigma-Aldrich, St. Louis, MO, USA), the membranes were incubated with an anti-phospho-Akt (p-Akt) primary antibody (Cat. No. STJ90166, St John’s Laboratory, London, UK, used at 1:1000) overnight at 4 °C. Following washing, the membranes were incubated with an appropriate horseradish peroxidase-conjugated secondary antibody (Cat. No. AS014, ABclonal, Woburn, MA, USA) at room temperature. Immunoreactive bands were visualized using a chemiluminescent substrate, and band intensities were quantified using Adobe Photoshop. β-Actin (sc-47778, Santa Cruz Biotechnology, Dallas, TX, USA) was used as the loading control. Three independent biological replicates (n = 3) were analyzed for each treatment condition.

### 4.9. Statistical Analysis

Statistical analyses were performed using GraphPad Prism version 9 (GraphPad Software, San Diego, CA, USA). Data distribution was assessed using the Shapiro–Wilk normality test, and homogeneity of variances was evaluated using the appropriate test. For the MTT, cell cycle and Annexin V analyses, which compared LA concentrations at a single treatment duration, we performed one-way ANOVA. For analyses involving both LA concentration and treatment duration, including colony formation, Ki-67 immunoreactivity, LC3-II immunoreactivity, RT-qPCR, migration, Western blot and invasion analyses, two-way ANOVA was performed using LA concentration and treatment duration as the two factors [43]. This factorial analysis evaluated the main effects of LA concentration and treatment duration, as well as the concentration × treatment duration interaction. Following ANOVA, Tukey’s multiple-comparisons post hoc test was performed for pairwise comparisons. Data are presented as mean ± standard deviation (SD). All statistical analyses were performed at a 95% confidence level, and *p* < 0.05 was considered statistically significant.

## 5. Conclusions

In conclusion, this study shows that LA exerts antiproliferative, cytotoxic, and antimigratory effects in Ishikawa endometrial cancer cells, characterized by reduced metabolic activity and clonogenic capacity, decreased Ki-67 expression after prolonged exposure, increased apoptosis, S-phase accumulation, and suppressed cell migration. These functional effects were accompanied by concentration- and time-dependent molecular changes, with prolonged LA exposure particularly associated with reduced Akt phosphorylation and mTOR transcript levels. In contrast, PI3K and Akt1 transcript levels and autophagy-related markers showed more heterogeneous patterns, highlighting the dynamic nature of the cellular response to LA. Collectively, these findings identify Akt/mTOR- and autophagy-related molecular alterations associated with the cellular effects of LA in Ishikawa cells without establishing a direct causal mechanism. Further studies using pathway-specific inhibition, autophagic flux analyses, additional endometrial cancer models, and in vivo approaches are warranted to define the underlying mechanisms and determine whether these findings extend beyond the Ishikawa cell model.

## Figures and Tables

**Table 1:** Forward (F) and reverse (R) primer sequences and lengths (bp) used for RT-qPCR analysis of β-actin, PI3K, mTOR, MAP1LC3B, and Akt1.

Primer	Sequences	Length (bp)
β-actin F	GCCAACTTGTCCTTACCCAGA	21
β-actin R	AGGAAGAGAGACTTGACCC	19
PI3K F	GGACCCGATGCGGTTAGAG	19
PI3K R	ATCAAGTGGATGCCCCACAG	20
mTOR F	TTCCGACCTTCTGCCTTCAC	20
mTOR R	CCACAGAAAGTAGCCCCAGG	20
MAP1LC3B F	GCACCAACCCACCTACTCTG	20
MAP1LC3B R	GTGGGTGAGGGGCAGATG	18
AKT1 F	GGAGGTTTTTGGGCTTGCG	19
AKT1 R	GTCCATGGTGTTCCTACCCA	20

## Data Availability

The original contributions presented in this study are included in this article. Further inquiries can be directed to the corresponding author.

## Abbreviations

The following abbreviations are used in this manuscript:

AEC	3-amino-9-ethylcarbazole
Akt	Protein kinase B
ANOVA	Analysis of variance
BSA	Bovine serum albumin
Cdna	Complementary DNA
DMEM	Dulbecco’s Modified Eagle Medium
DMSO	Dimethyl sulfoxide
EC	Endometrial cancer
EDTA	Ethylenediaminetetraacetic acid
FBS	Fetal bovine serum
FITC	Fluorescein isothiocyanate
ICC	Immunocytochemistry
IC_50_	Half-maximal inhibitory concentration
LA	Licochalcone A
LC3-II	Microtubule-associated protein 1 light chain 3-II
Mtor	Mammalian target of rapamycin
MTT	3-(4,5-dimethylthiazol-2-yl)-2,5-diphenyltetrazolium bromide
p-Akt	Phosphorylated Akt
PBS	Phosphate-buffered saline
PI3K	Phosphoinositide 3-kinase
RIPA	Radioimmunoprecipitation assay
RNA	Ribonucleic acid
RT-qPCR	Reverse transcription quantitative real-time polymerase chain reaction
SD	Standard deviation
SDS-PAGE	Sodium dodecyl sulfate-polyacrylamide gel electrophoresis
T-TBS	Tris-buffered saline containing Tween-20
7-AAD	7-aminoactinomycin D
